# Of mitochondrion and COVID-19

**DOI:** 10.1080/14756366.2021.1937144

**Published:** 2021-06-09

**Authors:** Khalid Omer Alfarouk, Sari T. S. Alhoufie, Abdelhameed Hifny, Laurent Schwartz, Ali S. Alqahtani, Samrein B. M. Ahmed, Ali M. Alqahtani, Saad S. Alqahtani, Abdel Khalig Muddathir, Heyam Ali, Adil H. H. Bashir, Muntaser E. Ibrahim, Maria Raffaella Greco, Rosa A. Cardone, Salvador Harguindey, Stephan Joel Reshkin

**Affiliations:** a Research Center, Zamzam University College, Khartoum, Sudan; b Department of Evolutionary Pharmacology and Tumor Metabolism, Hala Alfarouk Cancer Center, Khartoum, Sudan; c Al-Ghad International College for Applied Medical Sciences, Al-Madinah Al-Munwarah, Saudi Arabia; d Medical Laboratories Technology Department, College of Applied Medical Sciences, Taibah University, Al-Madinah Al-Munwarah, Saudi Arabia; e Faculty of Medicine, Al-Azhar University, Cairo, Egypt; f Assistance Publique, des Hôpitaux de Paris, Paris, France; g College of Applied Medical Sciences, Najran University, Najran, Saudi Arabia; h College of Medicine, University of Sharjah, Sharjah, UAE; i Department of Pharmacology, College of Pharmacy, King Khalid University, Abha, Saudi Arabia; j Pharmacy Practice Research Unit, Clinical Pharmacy Department, College of Pharmacy, Jazan University, Jazan, Saudi Arabia; k Faculty of Pharmacy, University of Khartoum, Khartoum, Sudan; l Institute of Endemic Diseases, University of Khartoum, Khartoum, Sudan; m Department of Biosciences, Biotechnologies, and Biopharmaceutics, University of Bari, Bari, Italy; n Institute for Clinical Biology and Metabolism, Vitoria, Spain

**Keywords:** COVID-19, mitochondrion, inflammation, cytokine storm, treatment

## Abstract

COVID-19, a pandemic disease caused by a viral infection, is associated with a high mortality rate. Most of the signs and symptoms, e.g. cytokine storm, electrolytes imbalances, thromboembolism, etc., are related to mitochondrial dysfunction. Therefore, targeting mitochondrion will represent a more rational treatment of COVID-19. The current work outlines how COVID-19’s signs and symptoms are related to the mitochondrion. Proper understanding of the underlying causes might enhance the opportunity to treat COVID-19.

## Introduction

COVID-19 is a new emerging pulmonary infection caused by SARS-COV-2. It is characterised by flu-like symptoms often followed by acute pulmonary inflammation. Multiple viruses are known to cause both inflammation and mitochondrial dysregulation (metabolic shifts). The influenza virus H1N1 targets the mitochondria of type II cells^1^. Multiple other inflammatory viruses are known to induce metabolic changes, such as the cytomegalovirus (CMV)^2^, the Epstein-Barr virus (EBV)^3^, or the hepatitis virus (HCV)^4^. These viruses interfere with cellular metabolism, increase glucose uptake, and decrease the mitochondrial energy yield resulting in intense glycolysis. In Caco-2 cells, infection with SARS-CoV-2 has been found to up-regulate carbon metabolism and decrease oxidative phosphorylation. I removed it because it is out of context and there is no reference- also no reference for the Caco-2 cells.

The mitochondrion is a doubled-membrane organelle, represents the backbone of the eukaryote cell metabolism^5^^,^^6^. Mitochondrion is the cells' metabolic generator and plays a significant role in determining cellular proliferation^7^, cellular death pathways^8^ and also plays a crucial role in maintaining the redox state of the cell^9^.

Many viral diseases disturb the mitochondrial physiology^10–12^, e.g. Epstein–Barr virus (EBV) affects mitochondrial fission^13^, herpes simplex virus type 1 (HSV-1) and pseudorabies virus (PRV) affect calcium homeostasis^14^, and many viruses, e.g. influenza viruses, Hepatitis B virus, support and/or encode proapoptotic proteins that lead to programmed cell death^15–17^.

Since the occurrence of unidentified pneumonia patients in Wuhan hospitals in China in late 2019 and the labelling of the disease by the World Health Organisation (WHO) as severe acute respiratory syndrome coronavirus 2 (SARS-CoV-2), the disease became a pandemic in less than three months, and as of the beginning of December 2020 the total confirmed cases of COVID-19 reached 65,257,767 worldwide according to a WHO update^18–20^.

Despite the increased global incidence records of the COVID-19 cases, most of the infected patients showed either mild infection with no fever or signs of pneumonia or moderate infection with clinical manifestations like cough, sore throat, fever ≥38 °C, fatigue, and shortness of breath^21^.

Severe infection with increased mortality rate occurs with pneumonia and respiratory failure. At the same time, other complications might present, such as acute respiratory distress syndrome (ARDS), microvascular thrombosis, coagulopathy, liver injury, acute kidney injury, acute cardiac failure and shock^22–27^. Factors affecting the infection’s severity are not fully understood; however, factors such as the state of the immune system, viral load, and underlying comorbid diseases might play a role in the severity of the infection^28–30^.

In the current work, we present COVID-19 as a mitochondriopathy and demonstrate that many of the hallmarks of COVID-19 are driven by mitochondrial injury.

## The role of mitochondria and cytokine storm

Hyperinflammation – e.g. cytokine storm – is a hallmark of COVID-19^31^. Such hyper-inflammation occurs due to a massive increase in Reactive Oxygen Species (ROS)^32^^,^^33^. Increased ROS results in the release of tumour necrosis factor (TNF)-α and interleukin-1β (IL-1β)^34^^,^^35^. The mitochondrion is a significant source of ROS in mammalian cells^36^. Therefore, the mitochondrion lies within the cytokine storm's core^37^.

The inflammasome is a cytosolic complex composed of multiple proteins of innate immunity to promote and activate the proinflammatory mediators such as IL-1β, IL-18^38–41^. One protein component is an intracellular pathogen sensor called nucleotide-binding oligomerization domain-like receptors, or NOD-like receptors (NLRs)^42^. NLRP3 is one NOD-like receptor (NLRs) family member that represents the backbone of the inflammasome. The role of NLRP3 in inflammation and the cytokine storm is crucial and complex. As a consequence of its activation, the cell reprograms its metabolic machinery into increased glycolysis with a subsequent reduction of the Krebs' cycle^43^, i.e. induces mitochondrial atrophy. ROS also activates the NLRP3 where it is associated with mitochondrial cardiolipin^40^ and might be correlated with mitochondrial ageing (which stimulates the inflammasome)^44^.

SARS-COV-2 infection attacks the mitochondrion, especially the phosphorylation (OxPHOS) pathway, e.g. Complex-I^45^, which results in abnormal ROS production supporting cellular diseases and ageing. SARS-CoV-2 might directly activate the NLRP3 inflammasome, with consequent flaring-up of the inflammation cascade^40^. Hence, SARS-COV-2 alters mitochondrial physiology^46^^,^^47^.

## COVID-19 disrupts the possible mitochondrial role in iron homeostasis

Iron is an essential nutrient and its levels differ from one tissue to another and also depend on the tissues pathological state^48^. Cellular iron homeostasis is a complexed process^49^, but generally, it could be described as: the entrance of iron to the cell through: (i) endocytosis of transferrin receptor 1 (TfR1), or (ii) ferrous iron (Fe^+2^) transporters e.g. divalent metal transporter 1 (DMT1)^50^ and Zinc transporters 8, 14 (ZIP8, ZIP14)^51^^,^^52^ with the assistance of the iron reductase enzyme Metalloreductase STEAP2^53^, Duodenal cytochrome B (Dcytb)^52^, and Stromal cell-derived receptor 2 (SDR-2)^54^. After being taken-up, the iron is stored in ferritin^55–57^ for different biochemical functions including the formation of ROS^58^^,^^59^ and managing transcription through regulating the iron-responsive element-binding proteins (IRP1, IRP2)^60^^,^^61^. After that, iron export from the cell occurs via ferroportin-1 (also termed as solute carrier family 40 member 1 (SLC40A1) or iron-regulated transporter 1 (IREG1))^62^.

The role of mitochondria in iron homeostasis is one of the most challenging of recently addressed issues. Generally, ferritin is an intracellular protein that can act as an iron-buffering agent to re-equilibrate iron deficiency or iron overload^63^. Ferritin is stored in the mitochondrion and imported from the cytoplasm via mitoferrin carriers^64^^,^^65^.

Disruption of mitoferrin leads to hyperferritinemia, accompanied by hyper-inflammation, an additional hallmark of COVID-19 severity^64^^,^^66^^,^^67^. Severe iron overload leads to mitochondrial DNA damage that exacerbates the cellular oxidative stress^68^.

For this reason, the iron-chelating agent, Deferoxamine, has been introduced in the management of COVID-19^69^^,^^70^.

## Lactate dehydrogenase in COVID-19

The lactate dehydrogenase (LDH) is an enzyme that catalyses a reversible biochemical reaction that converts pyruvate into lactate. After glucose entry, the hydrogen ions (proton, H^+^) level is rising, alters the cell's optimum pH to process its chemical pathways. After completing the Krebs' cycle, the cell yields in CO_2_, energy in ATP, and hydrogen ions. The oxygen reacts with H^+^ to produce water. Therefore, oxygen in cellular respiration acts as a detoxifying agent (acting as a buffer)^71^. During transient hypoxia, some tissues, e.g. heart, brain, kidney, are prone to damage.

In contrast, other tissues are slightly adaptable by expressing the lactate dehydrogenase enzyme to shift the cellular metabolism to prevent the Krebs' cycle. Therefore, the glucose utilisation after its entry ends up by forming lactic acid and furthering extracellular acidity via Monocarboxylate Transporters (MCTs)^72–74^. So, metabolic shifting to end in lactic acid will decrease the possible intracellular acidity and promote the extracellular acidity that exacerbates the cytokine storm as lactate is a signalling molecule that supports inflammation^75^^,^^76^.

The conversion of pyruvate to lactate is associated with the conversion of NADH to NAD^+^. Increasing of NAD^+^ level inhibits not only mitochondrial metabolism but also supports the inflammation process^77^^,^^78^.

LDH is correlated with COVID-19 and its severity^79^ because the lactate synthesis is increased. The level of blood lactate is a prognostic factor for the intensity of the lung's inflammation and decreased survival^80^.

### Dysregulation of calcium homeostasis during COVID-19 affects mitochondrial biology

Calcium is a vital electrolyte that plays many critical roles in cellular physiology^81^. Calcium governs intracellular mitochondrial motility (mitochondrial dynamics)^82^^,^^83^, manages mitophagy^84–86^, controls ATP production^87^, and impacts the role of the mitochondrion in the redox statue of the cell^88^.

A reduced level of calcium is well-documented in covid-19 infection, and it is thought to have a role in its poor prognosis^89^. Therefore, hypocalcaemia has a detrimental effect on the mitochondrion, promotes ROS formation, and activates the inflammatory cascade.

## The role of the mitochondrion on coagulability

### D-dimer

While the term D-dimer reflects the dimerisation process (two subunits), it also seems to be an erroneous name suggested by one of the researchers that discovered it^90^^,^^91^. All in all, D-dimer is fibrin fragments that are crosslinked with polypeptide bonds due to the degradation of fibrinogen via plasmin^92^^,^^93^. Higher levels of D-dimer in the blood represent a severe sign of thromboembolism^94–96^ and recently has become an indicator of how COVID-19 patients develop thromboembolism and the disease severity^97–99^ since D-dimer level is markedly increased among critical patients and is a significant risk factor for mortality^100^

Oxidative stress is associated with thromboembolism^101^, in that ROS activates urokinase plasminogen activator (UPA)^102^, subsequently producing plasmin that hydrolyses fibrinogen into D-dimer. The increased Plasmin, in turn, increases ROS^103^, which produces an out-of-control positive feedback between ROS and plasmin. Furthermore, D-dimer expression also might increase the level of urokinase-type plasminogen activator (plasmin activator), and so it also enters a vicious cycle producing thromboembolism.

There is an inverse relationship between functional mitochondrial and urokinase plasminogen, such that upregulation of the UPA is an indicator of reduced mitochondrial function while, in contrast, downregulation of UPA restores mitochondrial function (e.g. activation of programmed cell death)^103^.

### Troponins

These are a group of proteins found in the heart and skeletal muscle that mediate calcium-dependent muscle contraction^104^^,^^105^. An increased level of troponins in the blood is an indicator of necrosis rather than programmed cell death, i.e. mitochondrial injury or dysfunctionality due to hypoxia^106–112^.

COVID-19 is associated with higher troponin levels^113^, which might correlate with mortality^114^. Indeed, higher troponin levels were confined to cardiac disorder and other diseases, such as sepsis or renal disease^115^, both of which were correlated with COVID-19^112^^,^^116^^,^^117^. Also, during cardiac and muscle injury, troponin levels are increased significantly in severe disease patients, leading to progression towards multiple organ failure (MOF) and death.

## Targeting the mitochondrion to treat COVID-19

**Figure 1.**
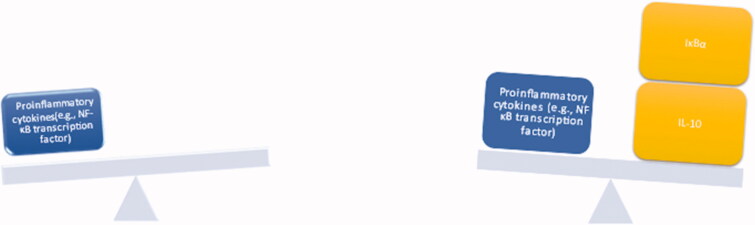
How does Amiloride re-equilibrate the cytokine storm via boosting the anti-inflammatory cytokines and suppressing the proinflammatory cytokines.

**Figure 2.**
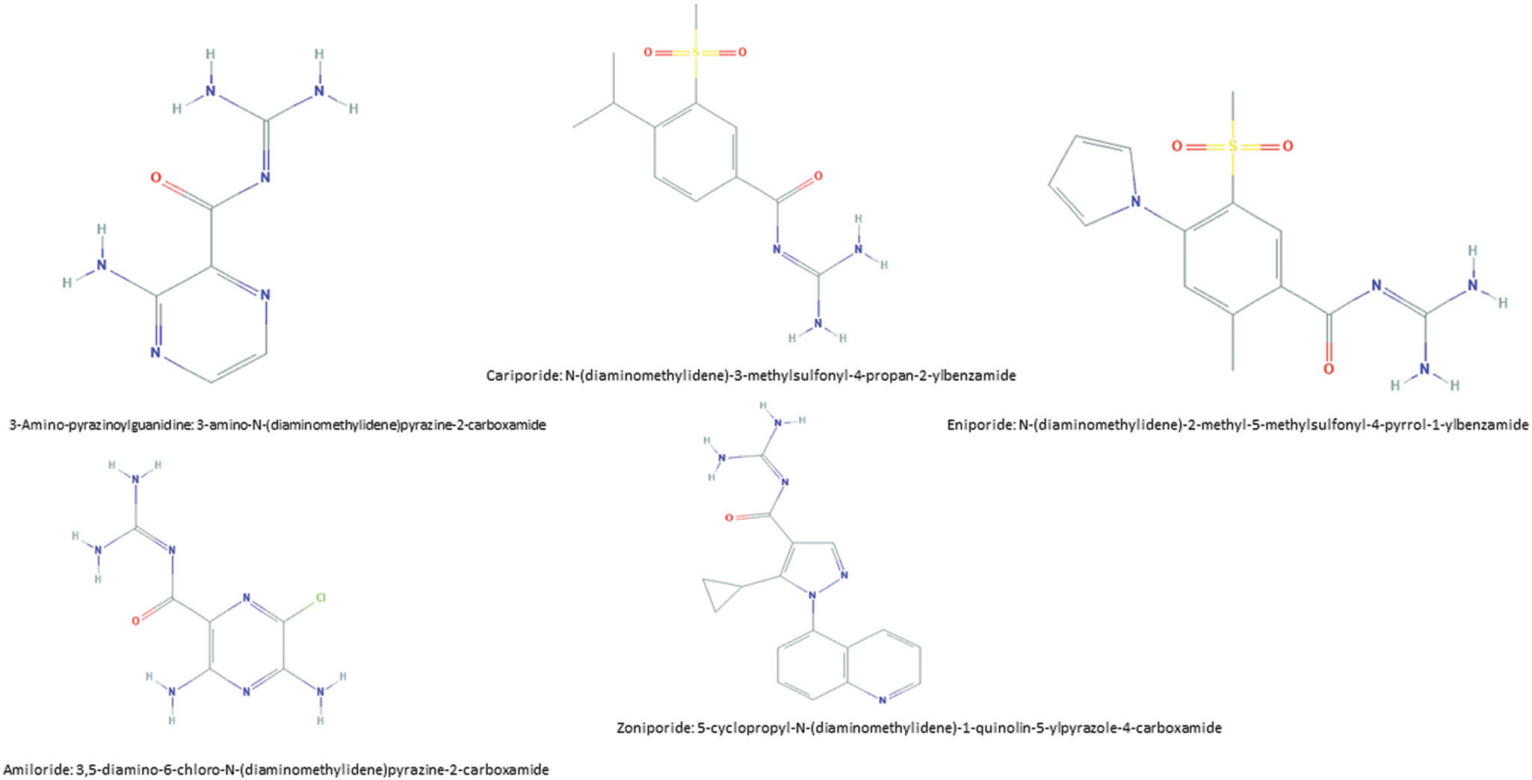
Different chemical formula of some of NHE1 inhibitors.

**Figure 3.**
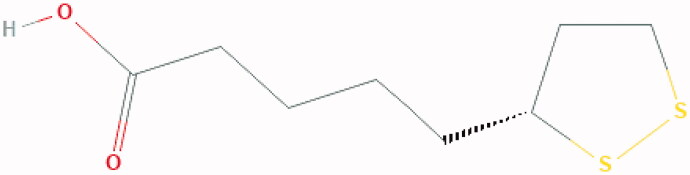
Chemical Structure of lipoic acid: 5-[(3R)-dithiolan-3-yl] pentanoic acid.

**Figure 4.**
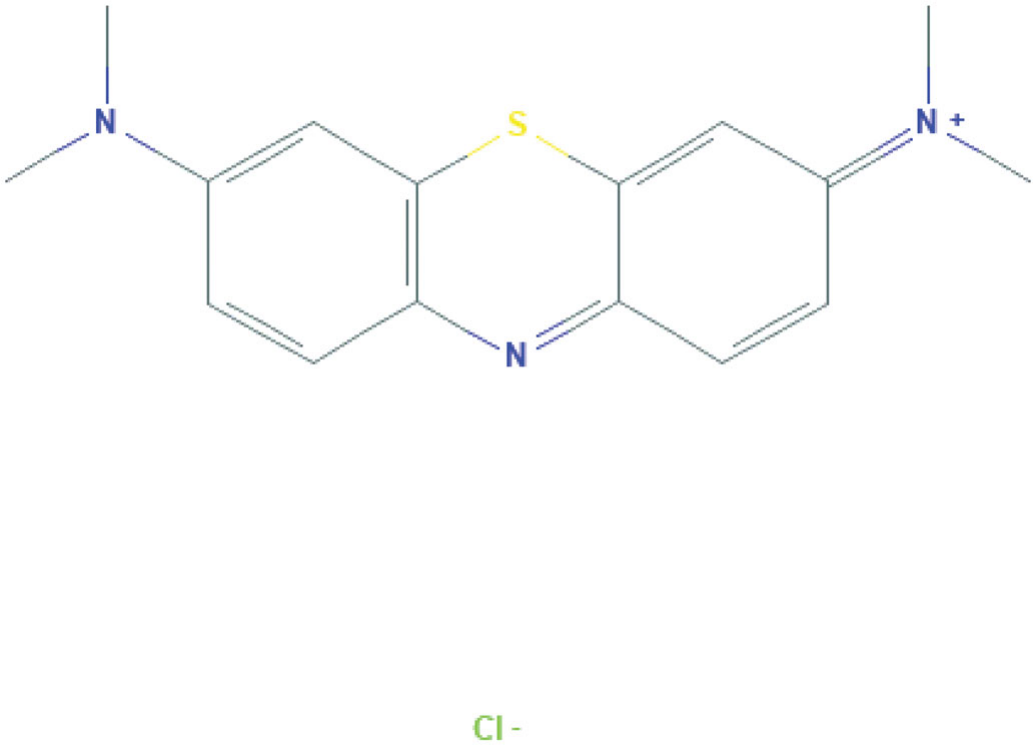
Chemical structure of methylene blue: [7-(dimethylamino) phenothiazin-3-ylidene]-dimethylazanium;chloride.

**Figure 5.**
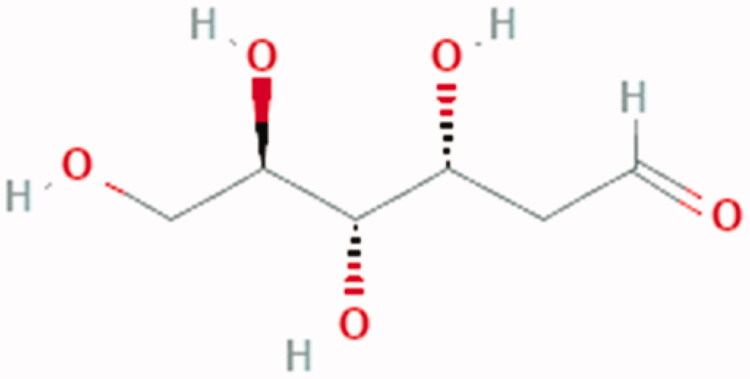
Chemical Structure of 2DG: (3 R,4S,5R)-3,4,5,6-tetrahydroxyhexanal.

In 1956, Otto Warburg suggested that cancer occurs due to mitochondrial injury and, in this respect, it seems that COVID-19 could be looked at as an extrapolation of cancer^118^. At least it could be analysed through Warburg's lens and could stimulate the debate of whether mitochondriopathy is a direct cause of COVID-19 via SARS-COV-2 infection or just a symptom of COVID-19 in which, at least, mitochondrial injury might represent an early step of the SARS-COV-2 disease cascade. In this regard, the administration of pharmacological and non-pharmacological modulators of mitochondrial function^119^ could enhance patient recovery and improve patients' quality of life and might boost the vaccine's efficacy in the aged population (mitochondrial is a hub of ageing). An example of those agents includes:

1. NHE1 inhibitors:
  - In 2000, Reshkin et al. observed that the over-expression of NHE1 is the first event of carcinogenesis followed by alkaline increases in intracellular pH (alkaline pH_i_)^120^^,^^121^; and alkaline pH_i_ results in mitochondrial atrophy. Therefore, NHE1 inhibition, and specifically mitochondrial NHE1, will boost the mitochondrial functionality^122^ and so decrease the effect of SARS-COV-2.
  - Amiloride is a potassium-sparing diuretic, and it is a well-known NHE-1 inhibitor. Amiloride perturbs SARS-COV-2 biology^123^, and early reports showed that Amiloride inhibited coronavirus replication^123^
  - Amiloride also has potential as an anti-cytokine storm agent^124^. One of the possible mechanisms of action that explains how Amiloride antagonises the cytokine storm via contrasting the effect of proinflammatory mediators (e.g. the NF-κB transcription factor), by boosting the expression of anti-inflammatory mediators such as Interleukin-10 (IL-10), and nuclear factor of kappa light polypeptide gene enhancer in B-cells inhibitor, alpha (IκBα)^124^ (see Figure 1).
  - Significantly, Amiloride also suppresses the urokinase plasminogen activator (UPA), which might have a promising role in preventing thromboembolism^125^^,^^126^ and also prevents heart ischaemia^127^other NHE1 inhibitors include Cariporide, Eniporide, etc. (see Figure 2).
2. Fermented wheat germ extract:
  - Fermented wheat germ extract (FWGE) is a dietary supplement used to treat cancer and to slow ageing. The mode of action of FWGE is a mitochondrial restoration agent as it modulates the activity of the pyruvate dehydrogenase (PDH) complex to support the production of ATP from mitochondria^128^. Also, FWGE inhibits LDH and reduces the NAD^+^ levels^128^. Moreover, it shows promising action as an anti-cytokine storm drug^129–131^.
3. α-lipoic acid:
  - The history of α-lipoic dates to the 1950s (Figure 3) when German industry developed this drug. The first use of α-lipoic acid was for peripheral neuropathy due to diabetes^132^.
  - A preliminary Chinese study suggests the efficacy of α-Lipoic acid in the treatment of COVID-19^133^, where α-lipoic acid might act in the same way as FWGE; combined with hydroxycitrate, it synergizes the effect as an acting buffer to correct pH_i_ to restore mitochondrial function^134^^,^^135^.
4. Methylene Blue
  - Methylene Blue is the oldest of synthetic drugs (Figure 4), even before aspirin. Heinrich Carro manufactured it in 1876 for the German firm BASF. Methylene blue is a simple molecule. The fusion of two benzene rings with one nitrogen and one sulphur atom leads to a tricyclic aromatic compound which has a complex pharmacology and multiple clinical indications. Its mechanism of action involves a stabilising effect on mitochondria. Also, Methylene blue inhibits the replication of SARS-CoV-2^136^ and we reported a cohort of patients treated for cancer by Methylene Blue in cases without SARS-CoV-2^137^.
5. 2-deoxy-d-glucose (2DG)
  - The German scientist Otto Warburg discovered the Warburg effect in the 1920s^138^. Warburg stated that cancer cells display increased glycolysis and lactic acid secretion and, opposite to normal cells, the presence of oxygen does not inhibit this fermentation. The advent of Positron Emission Tomography (PET) scan combined with radio-labelled fluorodeoxyglucose has revived interest in the Warburg effect as there is an increased uptake of labelled glucose in the primary tumour and its distant metastases. The Warburg effect explains some of the cancer's hallmarks^118^^,^^135^ shift to aerobic glycolysis that has been reported to stimulate cell growth, evade tumour suppression, and resist cell death^139^. Increased pressure resulting from unrelenting proliferation in the affected organ's limited space results in cells' extrusion in the vasculature and distant metastases. The release of lactic acid in the extracellular space is a consequence of the Warburg effect. Lactic acid promotes angiogenesis and immune cell modulation^140^.
  - Infection with SARS-CoV-2 in Caco-2 cells has been found to up-regulate glycolytic carbon metabolism and decrease oxidative phosphorylation. In line with this, treatment with the glycolysis inhibitor 2-deoxy-d-glucose (2DG) prevents replication of SARS-CoV-2 in these cells^141^ (Figure 5).
  - The Warburg hypothesis was based on mitochondrial injury, but the debate is whether it is a cause of malignant transformation or just a consequence. Irrespective of which is correct, mitochondrial damage supports evolutionary tumour trajectory^142^. Parallel to this context, COVID-19 is associated with mitochondrial injury and such injury supports SARS-COV-2 pathogenicity and confers its evolutionary advantage. However, a significant concern is whether COVID-19 patients will develop cancer in the future due to such mitochondrial injury?

## Recommendations and concluding remarks

**Figure 6.**
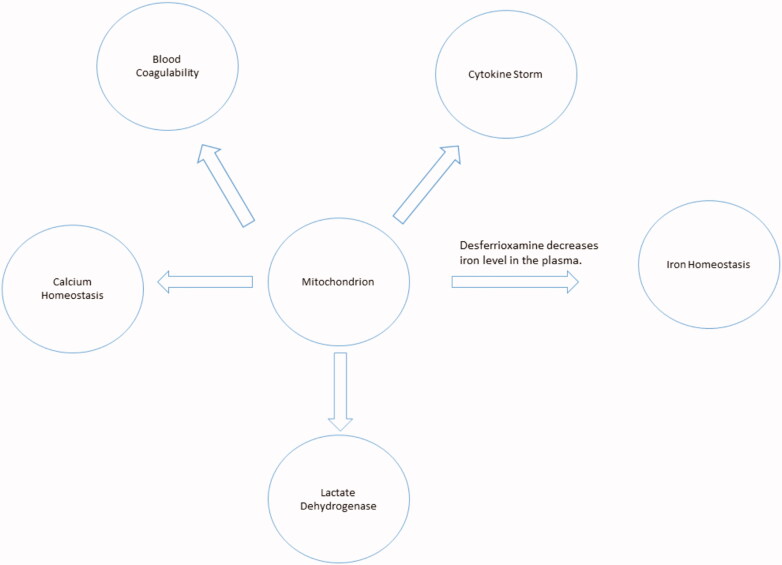
The mitochondrion lies within the core of COVID-19 cardinals.

COVID-19 has become a pandemic disease. The biology of the disease is exceptionally intricate, including many overlapping pathways. However, while the mitochondrion lies at the core of these pathways, its importance demands immediate attention and further investigation. A proper understanding of mitochondrial biology in COVID-19 pathogenesis will significantly enhance the strategy of fighting SARS-COV-2 (Figure 6). This paper has discussed and suggests a couple of pharmacological modulators that might represent potentially promising anti-COVID-19 treatments to block its progression and alleviate its aggressiveness.
